# Severe Anaphylaxis in Pregnancy: A Systematic Review of Clinical Presentation to Determine Outcomes

**DOI:** 10.3390/jpm11111060

**Published:** 2021-10-22

**Authors:** Anca Angela Simionescu, Bianca Mihaela Danciu, Ana Maria Alexandra Stanescu

**Affiliations:** 1 Department of Obstetrics and Gynecology, Filantropia Clinical Hospital, Carol Davila University of Medicine and Pharmacy, 050474 Bucharest, Romania; 2 Department of Obstetrics, Gynecology and Neonatology, National Institute for Maternal and Child Health “Alfred Rusescu”—Polizu, 127715 Bucharest, Romania; biamidan@yahoo.com; 3 Department of Family Medicine, Carol Davila University of Medicine and Pharmacy Carol Davila, 050474 Bucharest, Romania; alexandrazotta@yahoo.com

**Keywords:** anaphylaxis and pregnancy, hypoxic–ischemic encephalopathy, adrenaline

## Abstract

Anaphylactic reactions during pregnancy can range from subjective cutaneous symptoms to anaphylaxis and lethal anaphylactic shock. The fetal and maternal outcomes are unpredictable. This study is the first systematic review of the clinical presentation of severe anaphylaxis in pregnancy as defined by the World Allergy Organization to determine maternal and fetal outcomes. We searched PubMed, the Web of Science, and Scopus databases for articles published between 1 January 1985 and 15 April 2021 using the following terms (((anaphylactic shock) AND (pregnancy)) OR ((anaphylaxis) AND (pregnancy))). In 42 studies involving 47 patients, 36.17% of patients were 31–35 years old, and 74.47% of cases occurred peripartum, mostly during cesarean section. Accurate diagnosis with valid and reliable outcome measures was reported for 71.74% of cases. Twenty-two allergens were identified: antibiotics (penicillins and cephalosporins), anesthetic drugs (suxamethonium, mepivacaine), latex, oxytocin, sodium and sucrose iron, laminaria, misoprostol, rubber from Foley catheter, oral phytomenadione, ranitidine, chamomile, and ant sting. Two cases of maternal death related to latex and intravenous iron sucrose, and six infants with neurological disease were reported, mostly related to antibiotics. This review of the currently available literature shows that favorable outcomes are attainable with a high degree of observation, multidisciplinary cooperation, and rapid treatment.

## 1. Introduction

The diagnosis of an anaphylactic reaction, and determining the allergen responsible, is a clinically challenging situation. Anaphylactic reactions during pregnancy can range from subjective cutaneous symptoms to anaphylaxis and lethal anaphylactic shock. They are emergency situations requiring rapid diagnosis and management in obstetrics because of the unpredictable evolution from spontaneous resolution to serious maternal and fetal consequences, which may represent a life-threatening condition for both the mother and fetus, including severe neurological defects [1]. Based on European data, the reported incidence of anaphylaxis during pregnancy and labor varies from 1.44 to 2.7 cases per 100,000 births [2,3,4] depending on case definition. The case fatality rate for anaphylaxis in the general population is low, <0.001% [5], but the specific anaphylaxis-related maternal mortality ratio during pregnancy is estimated to be 0.09 per 100,000 live births [3].

The events may be underdiagnosed, as no consistently obvious signs and symptoms suggest improvement or lethality. Severe forms during pregnancy and labor are difficult to differentiate from severe hypotension due to spinal and epidural anesthesia, cardiopulmonary distress, amniotic fluid embolism, or placental abruption. The symptoms of anaphylactic shock may also involve subtle signs common for pregnancy, such as lower back pain, vulvar and vaginal itching, fetal distress, or premature birth [6]. Recent studies have also shown that painful uterine contractions can be a form of anaphylactic shock in both pregnant and non-pregnant women, but this manifestation is not frequently associated [7].

The literature on anaphylaxis during pregnancy was previously analyzed up to 2011 [8,9,10]. These reviews provided valuable insights into the general consideration of and data and guidance on diagnosis management and prevention, as well as awkward case reports. In addition, anaphylaxis-related maternal mortality in the obstetric setting is available from national or hospital data [2,3,4,11,12]. A recent systematic review of national databases covered maternal mortality and morbidity data highlights the need for guidelines and public health actions, including the diagnosis and management of anaphylaxis during pregnancy [13]. Because the total serum tryptase is only used to support an accurate diagnosis, we previously published the claim of biomarker utility to assess high-risk populations further; also, stratification of severity and risk prediction, necessitating additional exposure to potential anaphylactic triggers during obstetrical procedures [14]. Studies on the surveillance of adverse reactions to allergens are also available [15,16,17,18], and the topic is important for obstetricians, anesthesiologists, midwives, and allergist-immunologists, and family doctors.

There is a need for targeted actions and decisional markers to further assess this high-risk population, based on previous multicenter real-world clinical experience. We conducted a systematic review of clinical features and presentation fulfilling the World Allergy Organization criteria for anaphylaxis during pregnancy [19] to determine pregnancy outcomes after a reaction. We also aimed to describe the management of cases from the literature and related consequences for both the mother and infant.

## 2. Materials and Methods

### 2.1. Search Strategy

Our systematic review is based on a prospectively registered protocol (PROSPERO ID276244 [20]. This systematic review was initiated starting from the adapted Preferred Reporting Items for Systematic Reviews and Meta-Analyses (PRISMA) checklist adapted for case reports [21]. We used PubMed, the Web of Science, and Scopus databases for the literature search to find articles published between 1 January 1985 and 15 April 2021. We focused our search on articles published in English, French, and German. The search strategy was the same for all three databases and was based on the following terms: (((anaphylactic shock) AND (pregnancy)) OR ((anaphylaxis) AND (pregnancy))).

For the maternal case definition, we used clinical criteria for anaphylaxis as proposed by the World Allergy Anaphylaxis guidelines [5]: (1) acute onset of an illness (minutes to several hours) with simultaneous involvement of the skin, mucosa, or both (e.g., generalized hives, pruritus or flushing, swollen lips/tongue/uvula) and at least one of the following: respiratory compromise (e.g., dyspnea, wheeze/bronchospasm, stridor, reduced peak expiratory flow, hypoxemia), reduced blood pressure or associated symptoms of end-organ dysfunction (e.g., hypotonia (collapse), syncope, incontinence), and/or severe gastrointestinal symptoms (e.g., severe crampy abdominal pain, repetitive vomiting), especially after exposure to non-food allergens; or (2) acute onset of hypotension, bronchospasm, or laryngeal involvement after exposure to a known or highly probable allergen for that patient (minutes to several hours), even in the absence of typical skin involvement.

### 2.2. Study Selection

A three-stage study selection process was used. First, all titles and abstracts were initially screened for potential relevance by A.A.S and A.M.A.S independently. After the duplicates were removed, AAS and AMAS read the abstracts to choose articles that reported clinical data on patients as described below. The full texts of potentially relevant references were then screened.

Anaphylaxis severity included Grade 1 to 5 depending on clinical manifestations and organ involvement. We included original articles, reviews, case reports, and case series with a complete description of events from the administration of the potential allergen (no food allergen) to a description of the clinical course of the severe anaphylactic reaction (Grade 3–5), symptoms, signs, interventions, and pregnancy outcome. We also included cases diagnosed during pregnancy with clinical involvement of at least the respiratory tract, maternal hypotension, and/or tachycardia.

We limited inclusion to clinical cases that fulfill the World Allergy Organization criteria for symptomatology of anaphylaxis during pregnancy, cases that reported the name and time of administration of the allergen thought to be the cause of the anaphylaxis, as well as maternal evolution, management, and outcome. The etiological cause of anaphylaxis must have been reported as being a drug exposure, with the drug exposure occurring prior to the development of the signs of anaphylaxis. The time between the initiation of drug administration and the first clinical sign must have been reported and was limited to minutes and hours after exposure. Diagnoses had to be confirmed by laboratory tests during the event, including serum tryptase levels, IgE-specific allergen, or a basophil activation test, skin prick tests, or IgE tests if performed sometime after the event. For all cases included, we made the diagnosis independent of the diagnosis made by the authors.

We excluded cases due to food allergens, cases with a history of mast cell (MC)disorders, autoimmune diseases with previous anaphylactic manifestations, pseudocholinesterase deficiency, or exercise- or progesterone-induced anaphylaxis. We excluded conference reports, articles that described or commented on cases communicated by other authors, and articles describing cases published previously by the same team.

Data were exported into Microsoft Excel as follows:

- Author, year of publication, country of the report;
- Maternal age, parity, gestational age of the pregnancy, history of allergy, underlying comorbidities, maternal manifestations of anaphylaxis, management and outcomes including evolution, remission, and discharge home;
- Reported allergen of association, including dosage and time to onset of the first symptom of anaphylaxis;
- Criteria for severe anaphylactic reaction diagnosis, including clinical symptoms, presence of elevated tryptase levels, IgE, or other laboratory test confirming the diagnosis;
- Data about pregnancy course, labor, or cesarean section, including the type of anesthesia, the management of anaphylaxis by the anesthetist, and the management of obstetric complications;
- Infant data, if available, including sex, Apgar score, cord blood pH, the necessity of reanimation, immediate complications, neurological sequelae, and follow-up.

### 2.3. Assessment of Study Quality

To evaluate the methodological quality of selected cases, we used a score from 1 to 8 based on the tool proposed by Murad et al. [22]. The score is based on four domains: selection of patients so that the case can be generalized, ascertainment of exposure and outcome, causality and reporting sufficient data, and follow-up. Eight questions aid evidence-based practitioners and systematic reviewers in their assessment.

## 3. Results

**Figure 1:**
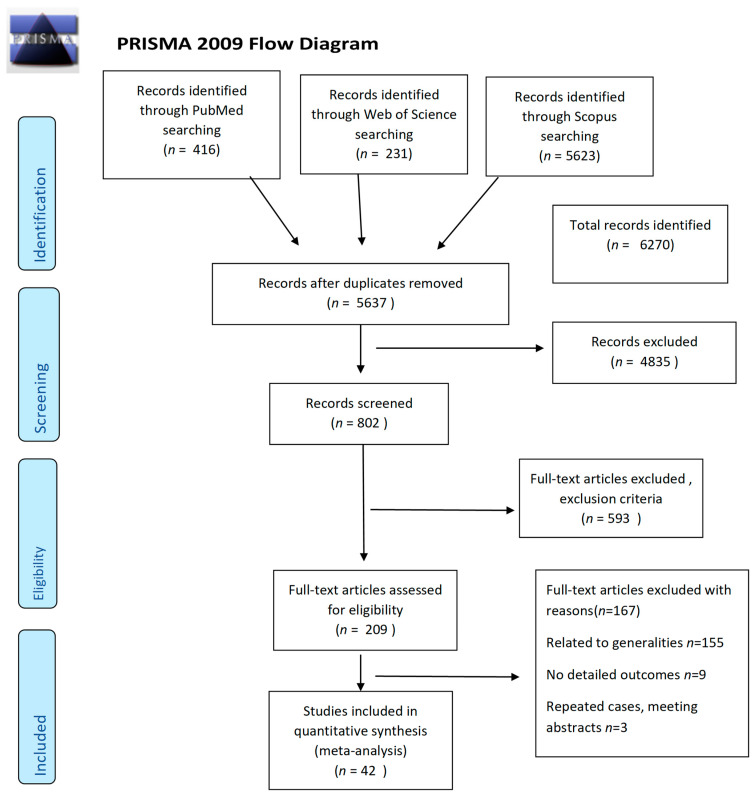
PRISMA flowchart of the literature review and selection process for clinical presentation and features of severe anaphylaxis in pregnancy to determine outcomes.

We analyzed 42 articles that met our criteria [7,8,10,15,20,23,24,25,26,27,28,29,30,31,32,33,34,35,36,37,38,39,40,41,42,43,44,45,46,47,48,49,50,51,52,53,54,55,56,57,58,59,60,61]. The search strategy is presented in Figure 1 using the PRISMA 2009 guidelines [21].

A total of 47 patients were analyzed, representing 22 allergens [10,15,20,28,29,30,31,32,33,34,35,36,37,38,39,40,41,42,43,44,45,46,47,48,49,50,51,52,53,54,55,56,57,58,59,60,61,62,63,64,65,66]. We found complete data from case reports and cases studies. Reports from Europe were most common (54.76%), followed by America (19.05%), Asia (21.43%), and Oceania (4.76%). Cases were published mostly in obstetrics/gynecology journals (16, 38%) and anesthesiology journals (14, 33.33%).

The overall quality of the cases was poor to moderate (Supplementary Materials Table S1). Accurate diagnosis with valid and reliable outcome measures was reported for 71.74% of cases.

Patient characteristics are presented in Table 1.

Thirty-five cases (74.47%) occurred peripartum, six cases (12.77%) presented with anaphylaxis during abortion induction, and six pregnant women (12.77%) experienced anaphylaxis during the management of co-pathologies during pregnancy (high risk of prematurity, condylomas destruction, pilonidal abscess, hyperemesis gravidorum, etc.). Anaphylaxis occurred in sixteen cases during scheduled cesarean section, in three cases during emergency cesarean section for obstetric indications, and in five cases before the onset of labor.

Case descriptions are presented in the Supplementary Materials (Tables S2 and S3). A negative history of atopy after anamnesis was noted in four cases after the occurrence of anaphylaxis; for latex allergy in case 2 with two prior cesarean sections without latex anaphylaxis, and in cases 18, 20, and 42 with previous use of laminaria without allergic reactions.

We found ten cases (21.27%) after antibiotic administration (n = 7 penicillin, n = 3 cephalosporin). Antibiotics were given in two cases for clinical chorioamnionitis, in two cases prophylaxis for streptococcal B, and in six cases prophylaxis for premature rupture of membranes or before cesarean section. Eleven cases (23.40%) were related to anesthetics: six cases after induction of general anesthesia with suxamethonium or rocuronium-sugammadex, two cases after vascular filling with colloidal solutions (dextran and volplex), and three cases with bupivacaine administration. Four cases of anaphylaxis occurred after the induction of abortion with cervical insertion of laminaria. Latex was responsible for anaphylaxis in nine cases (19.57%), including one case in which latex was associated with oxytocin. Other cases were related to rubber from a Foley catheter, misoprostol, intravenous use of iron sucrose, sodium ferric gluconate complex, ranitidine, phytomenadione oral, chamomilla, and pyridoxine. An ant sting by *Pachycondyla sennaarensis* was reported in one case of anaphylaxis.

The main clinical maternal symptoms related to specific allergens, maternal management, biochemical tests, and maternal outcomes from clinical cases are presented in Table 2.

Maternal oxygen and complex reanimation measures were used in all cases. Eleven cases with rapid cardiovascular shock and three cases with maternal cardiac arrest were reported. Two cases were reversible after external cardiac massage and electrical pulses. Two maternal deaths due to anaphylaxis were reported, one case related to latex leading to intravascular coagulation, and one occurred after iron sucrose administration. Two cases with intrapartum intravascular coagulation and one case of rhabdomyolysis post-event were reported.

Fetal bradycardia or deceleration on cardiotocography was reported after anaphylaxis for 20 cases (62.5%) among 32 cases of anaphylaxis occurring during labor or before preparing for cesarean section. In one case with fetal bradycardia at full dilatation (case 1), the patient gave birth vaginally after 10 min of anaphylaxis to a baby with metabolic acidosis at birth but normal neurological development at 9 months. In three cases (#6, 18, and 21), the fetal heart rate returned to normal, and the patients delivered vaginally, in all cases without fetal acidosis or hypoxemia. Neonatal neurological disease, including hypoxic encephalopathy, persistent hypotony, the rigidity of the extremities, and pyramidal syndrome, was reported in 6 out of 15 (40%) cases occurring after emergency cesarean section and fetal bradycardia after maternal anaphylaxis. Low Apgar score and hypoxia with acidosis were reported in 12 out of 20 (60%) cases. Three cases of fetal death were reported (cases 3, 12, and 13) with maternal anaphylaxis after oral phytomenadione, ant sting, or enema with an oily extract of chamomile flowers (Table 3).

Favorable fetal outcomes were reported in almost half of the cases after prompt management of the situation and emergency cesarean delivery in cases with fetal bradycardia or non-reassuring cardiotocography.

More than half (57.45%) of diagnoses of anaphylaxis based on clinical symptoms were confirmed by immediate serological tests. Prick skin tests and IgE antibody assays were performed 4–6 weeks after the anaphylactic reaction for 59.57% of cases. Differential diagnosis was maternal myocardial infarction or pulmonary embolism from trophoblastic disease and aortic dissection.

## 4. Discussion

To our knowledge, this is the first systematic review of the clinical presentation of anaphylaxis during pregnancy to determine maternal and fetal outcomes based on diagnosis, intensive care unit treatments, and follow-up. Though the complication of anaphylaxis during pregnancy can be life-threatening, a high degree of vigilance combined with prompt multidisciplinary management may achieve favorable maternal and fetal outcomes. The available real-world data quality is poor, mainly cases from obstetric and/or anesthetic settings of anaphylaxis and related maternal deaths based on national data [2,3,4]. The quality of studies is poor to moderate, as calculated in the Supplementary Material, but the case selection was adapted to this related clinical situation. The difficulty of these cases lies in the fact that they are rare, and at the time of the acute clinical manifestation, the management of the mother and fetus is life-saving. The mortality rate associated with anaphylaxis during pregnancy was estimated at 5% [3], and data from a national report from the UK found delays in diagnosis or misdiagnosis and inadequate follow-up during and after pregnancy. Gaps in management are inextricably linked to a need for coordinated, multidisciplinary care [62].

The allergen was retrospectively confirmed for all cases. This systematic review confirmed that, though the etiology varies, the most common cause of anaphylactic shock is the administration of antibiotics, followed by anesthetic medication [9,63]. Antibiotics were primarily administered prophylactically during cesarean section or for premature or prolonged membrane rupture and the prophylaxis of group B streptococcal infection. Iron deficiency is a common finding during pregnancy and may increase the risk of postpartum hemorrhage and puerperal sepsis [64]. Guidelines recommend oral iron therapy due to anaphylactic reactions; careful intravenous iron therapy is indicated when there is absolute non-compliance with, or intolerance of, oral iron therapy, proven malabsorption, or when a rapid Hb response is required [64]. Our review found two cases (cases 22 and 33) of anaphylaxis related to intravenous iron therapy, including one maternal death. Although a personal history of allergy or previous exposure to an allergen is considered a risk factor for anaphylaxis in obstetric and gynecologic settings [65], there are no data regarding the number of exposures. However, childbirth has been identified as the main cofactor for anaphylaxis in pregnancy, in our study 36.17% of anaphylaxis occurred during labor or scheduled cesarean section.

The variety of etiopathogenic factors and clinical manifestations of anaphylactic shock during pregnancy and peripartum is clear in this summary of existing studies. Before the onset or during labor, the most frequent differential diagnoses are amniotic fluid embolism or other causes of hypotension. It justifies the need for more validated decisional markers and a treatment protocol. In cases of collapse assumed to be due to anaphylaxis, the increased MC tryptase levels 1–4 h after the event can be useful in confirming the diagnosis [66,67], as well as serum allergen-specific IgE tests, and the diagnosis will be retrospectively confirmed [68,69]. During anaphylaxis, clinical laboratory tests may be challenging, as a normal tryptase level has been reported in up to 50% of patients with perianesthesia hypersensitivity reactions [70] and negative skin prick tests [19]. Elevated tryptase levels can be used to confirm the immunological mechanism of anaphylaxis, specifically MC activation and IgE-mediated allergic reactions. The optimal time to draw a serum tryptase level is 1–4 h after the acute event and to compare it with a control specimen taken at least 24 h afterward. False-negative tryptase levels have been reported, so skin testing should be strongly encouraged if clinical suspicion is high [55]. Despite clinical signs suggestive of anaphylaxis, high serum tryptase levels were reported for 57.44% of cases in this systematic review, and cutaneous tests were performed at a distance from the event in 29 cases.

A recent European multicenter study on anaphylactic shock in 63 pregnant women from 2012–2015 [3,4] found a great proportion of anaphylaxis during cesarean sections and after antibiotic and anesthetic drug administration, which is the same distribution as in the cases from the literature published between 1985 and 2021. Two women died in this recent report, compared to two maternal deaths between 1985 and 2021.

The treatment of anaphylactic shock begins by rapidly stopping the administration of the substance that triggered it and preserving the patient by preventing hypoxia by administering 100% oxygen, ventilatory support, and bronchodilators, managing hypotension by administering liquids in a large volume as well as adrenaline and prescribing treatment with antihistamines and corticosteroids. In addition, it is important to position the pregnant woman in the left lateral decubitus to promote blood flow to the heart and fetus [10,71]. All cases in this review had ventilatory support and the administration of a large volume of liquids. Corticosteroids and antihistamines were used when the supposed trigger was antibiotics or an anesthetic drug. Adrenaline was used when the first manifestation was hypotension or cardiovascular shock.

There is no consensus for these clinical cases regarding the definition of anaphylaxis. In these cases, the same management is applied to any subject suffering from this acute event. Evidence in the literature suggests that a poor outcome from anaphylaxis in the general population is associated with the late administration of adrenaline [72,73]. Adrenaline (epinephrine) administered intramuscularly as the antihypotensive agent of choice for maternal hypotension after spinal anesthesia [74] is a controversial choice in the treatment regimen in cases of anaphylactic shock during pregnancy. This is a potent sympathomimetic that affects both the alpha and beta-adrenergic receptors. The positive inotropic and chronotropic effects cause blood pressure to increase. For women, the most common side effects are arrhythmias, myocardial infarction, and intracranial hemorrhage [75]. There are also adverse effects on the fetus, caused mainly by uterine vasoconstriction, which predisposes it to hypoxia. In our review, the differential diagnosis was myocardial infarction in one case (case 28), but epinephrine was administered during maternal resuscitation.

Adrenaline has been studied in pregnant rhesus monkeys and is shown to be frequently associated with fetal bradycardia and acidosis. However, this effect has not been found in the administration of catecholamines directly into the fetal circulation [76]. In humans, the adverse effects caused by using epinephrine to treat anaphylactic shock in pregnant women were first recorded in the literature in 1984 when 1.5 mg of epinephrine was administered during a severe episode of hypotension. The newborn had severe neurological disorders, which demonstrated the fetal risk of catecholamines [77]. In our review, adrenaline was administered in five out of six cases with infant neurological sequelae and for three out of four cases of stillbirth. Interestingly, in one case (case 19), adrenaline was continued during labor after remission of anaphylaxis, but the baby was delivered vaginally without hypoxia or acidosis and had a favorable outcome.

Based on these findings, ephedrine has been proposed as a substitute for adrenaline during pregnancy due to its predominant beta-adrenergic effect leading to weaker uterine vasoconstriction. However, adrenaline remains an option due to its more potent effect with a faster resolution of maternal hypotension, which ultimately leads to better infusion of the uteroplacental territory [41].

The strength of this systematic review is that it provides the best available data with which to make a clinical decision about the drugs that may more likely be implicated as a trigger for anaphylactic reactions, the clinical management of pregnant patients undergoing anaphylaxis, and maternal and fetal outcomes. However, this review has several limitations, primarily due to the different time periods of reported cases from different countries and summary descriptions of the clinical cases. The data on the number of cases is of moderate to poor quality and there is a lack of data about pregnancy monitoring and differential diagnosis of fetal brain disease (e.g., intrauterine growth restriction, genetic or metabolic syndrome).

## 5. Conclusions

This review of the available literature shows that after anaphylactic reactions during pregnancy, favorable maternal and fetal outcomes are attainable with a high degree of vigilance, multispecialty cooperation, and rapid treatment. However, the team must be aware of subtle clinical manifestations, such as uterine contractions, vaginal itching, or lower back pain. The management of pregnant women with anaphylactic shock remains challenging, and rapid laboratory investigations and a standard protocol for the management and confirmation of the causative allergen are important. A deeper understanding of the mechanisms of anaphylaxis during pregnancy as well as targeted systems biology and proteomic and multi-omic approaches may provide a precise and accurate diagnosis, enabling forthcoming clinical features to be introduced in guidelines for anaphylaxis during pregnancy.

## Figures and Tables

**Table 1:** Distribution of 47 patients with severe anaphylaxis in 42 studies.

Characteristic	Case Number n (%)
Patient age, years	
15–20	4 (8.51)
21–25	5 (10.64)
26–30	12 (25.53)
31–35	17 (36.17)
36–40	6 (12.77)
>40	3 (6.38)
Parity	
Nulliparity	29 (61.70)
Primiparity	5 (10.64)
Second parity	6 (12.77)
>3 deliveries	7 (14.89)
History of atopy and allergy	
Yes	11 (23.40)
No	24 (51.07)
Not specified	12 (25.53)
Anaphylaxis timing	
During the first and second trimester *	9 (19.15)
During labor	
-spontaneous labor	10 (21.28)
-before labor or labor induction	10 (21.28)
During scheduled cesarean section	18 (38.29)
Clinical manifestation	
Hypotension	47 (100)
Tachycardia	47 (100)
Urticaria	27 (57.45)
Facial and glottic edema	14 (29.79)
Dyspnea	14 (29.79)
Stridor	12 (25.53)
Pruritus	9 (19.15)
Digestive symptoms	4 (8.51)
Generalized edema	3 (6.38)
Cyanosis	3 (6.38)
Thoracic pain	2 (4.26)
Agitation	1 (2.13)

* Occurrence during pregnancy, not related to childbirth (i.e., not when hospitalized for hyperemesis gravidorum, cervical cerclage, abortion induction, or condyloma destruction).

**Table 2:** Studies included in the systematic review of clinical features and symptoms of anaphylaxis in pregnancy and outcomes.

Triggers	Author, Year (Case Number)	History of Allergy/Previous Exposure When Is Mentioned	The Time between Exposure to the First Symptom	Core Maternal Outcome		Causality	
				Evolution ^a^	Discharge ^b^, Days	Biochemical Immediate Tests	Skin Tests at Distance
Antibiotics (β-lactam, cephalosporin)	Gallagher et al., 1988 [23] (1)	No/Yes	5 min	Few days	4	M	M
	Heim et al., 1991 [26] (4)	No/No	5 min	NS	NS	M	M
	Konno et al., 1995 [30] (8)	No	5 min	2 days	13	M	M
	Gei et al., 2004 [46] (41)	No/No	20 min	24 h	2D	M	M
	Berardi et al., 2004 [6] (23)	Yes/No	Seconds	NS	NS	M	M
	Khan et al., 2008 [46] (27)	Yes	Seconds	NS	6	M	M
	Chaudhuri et al., 2008 [10] (28)	No	Minutes	24 h	2	M	M
	Sleth et al., 2009 [8] (35	No/No	Minutes	NS	NS	+	+
	Göktaṣ et al., 2010 [51] (36)	No/No	One minute	2 h	NS	M	M
	Jeon et al., 2018 [58] (44)	No/No	Seconds	NS	NS	M	+
Anesthetic agents							
Suxamethonium, fentanyl/Suxamethonium	Edmondson et al., 1994 [29] (7)	No	10 min	NS	NS	+	+
	Stannard et al., 2001 [37] (17)	Yes	Minutes	NS	~28	+	+
	Rocchiccioli et al., 2009 [49] (32)	No/No	Minutes	NS	NS	+	+
	Sleth et al., 2009 [8] (34)	No/No	Seconds	NS	NS	+	+
	Truong et al., 2015 [61] (42)	No/No	Immediate	Extubated after 24 h	NS	+	+
Roncuronium-sugammadex complex	Yamaoka et al., 2017 [57] (43)	No/No	Hours	NS	NS	+	+
Dextran	Vatsgar et al., 2006 [44] (25)	No/No	Seconds	Extubated after 32 h	NS	+	M
Volplex	Karri et al., 2009 [50] (33)	No/No	Seconds	NS	NS	+	+
Mepivacaine	Takahashi et al., 2019 [60] (46)	Yes/No	Seconds	NS	NS	+	+
Oxytocin and latex	Jorrot et al., 1989 [21] (2)	No/No	15 min	NS	8	+	+
	Laurent et al., 1992 [27] (5)	Yes/Yes	Few minutes	2 h	NS	+	+
	Liccardi et al., 2013 [53] (38)	Yes/Yes	Minutes	NS	NS	+	+
Laminaria	Cole et al., 2000 [36] (16)	No/No	5 min	1day	~7	NA	NA
	Knowles et al., 2002 [40] (18)	No/Yes	30 min	Few hours	NS	NA	NA
	Kim et al., 2003 [42] (20)	No/Yes	30 min	NS	NS	NA	NA
	McQuade et al., 2020 [61] (47)	No/No	Seconds	NS	1	NA	NA
Misoprostol	Béné et al., 2014 [54] (40)	No/No	Less than 60 min	NS	2	+	NA
	Schoen et al., 2014 [55] (41)	No/No	Seconds	Extubated after 24 h	3	+	NA

^a^ Time to normal evolution; ^b^ Time to discharge home in days. NS, not clearly specified; NA, not attributed; M, missing.

**Table 3:** Newborn outcomes for anaphylaxis in pregnancy.

Stage of Pregnancy		Author, Year (Case Number)	Triggers	Delivery Outcome	Fetal Outcome
Second trimester		Vatsgar et al., 2006 [44] (25)	Dextran	CS after one month for preeclampsia	Favorable outcome
		Truong et al., 2015 [56] (42)	Suxamethonium	LI	Stillbirth
Third trimester	Before labor	Edmondson et al., 1994 [29] (7)	Suxamethonium, fentanyl/Suxamethonium	Emergency CS	Hypoxia and acidosis at birth. Pulmonary hemorrhage and neurological impairment.
		Rizk et al., 1998 [34] (14)	Ant sting	LI	Fetal Intrauterine death (placental abruption).
		Eckhout et al., 2001 [38] (18)	Latex	Vaginal delivery after 1 months	Favorable outcome.
		Shingai et al., 2002 [39] (19)	Latex	Emergency CS	Hypoxia and hypoxemia. Neurological impairment with rigidity.
		Cuciti et al., 2005 [43] (24)	Sodium ferric gluconate complex	Vaginal delivery after one week	Favorable outcome.
		Sleth et al., 2009 [8] (34)	Suxamethonium	Emergency CS	Hypoxia and hypoxemia. Favorable outcome.
		Sleth et al., 2009 [8] (35)	Amoxicillin	Emergency CS	Hypoxia and hypoxemia. Favorable outcome.
		Schoen et al., 2014 [55] (41)	Misoprostol	Emergency CS	Favorable outcome.
	Before Scheduled CS	Stewart et al., 1995 [31] (9)	Rubber	CS	Hypoxemia, acidosis, convulsions. Favorable outcome.
		Jeon et al.,2018 [58] (44)	Cefotetan	Emergency CS	Hypoxia, acidosis. No follow-up.
		Takahashi et al., 2019 [60] (46)	Mepivacaine	Emergency CS	Hypoxia and acidosis for both twins. Favorable outcome.
	During emergent CS in labor for obstetrical reasons	Stannard et al., 2001 [37] (16)	Suxamethonium	CS	Acidosis and hypoxemia. No follow-up.
	During labor	Gallagher et al., 1988 [23] (1)	Penicillin	Spontaneous delivery	Hypoxia and acidosis at birth. Favorable neurological outcome at 9 months.
		Anderson et al., 1989 [25] (3)	Phyto menadione (oral)	Emergency CS	Hypoxia and atonia. Baby died 6 h after birth.
		Heim et al., 1991 [26] (4)	Ampicillin	Emergency CS	Hypoxia and acidosis at birth. Neurological disease at 28 days and 6 months.
		Powell et al., 1993 [28] (6)	Ranitidine	Spontaneous delivery	Favorable outcome.
		Konno et al., 1995 [30] (8)	Cefazolin	Emergency CS	Hypoxia and acidosis. Favorable outcome at 12 months.
		Jensen-Jarolim et al., 1998 [35] (15)	Extract of chamomile flowers	IL	Stillbirth.
		Gei et al., 2004 [41] (21)	Ampicillin	Vaginal delivery	Favorable outcome.
		Berardi et al., 2004 [6] (23)	Ampicillin	Emergency CS	Hypoxia and acidemia. No follow-up.
		Khan et al., 2008 [46] (27)	Ceftriaxone	Emergency CS	Hypoxia and acidosis. Encephalopathy, hypoxia, cerebral ischemia.
		Chaudhuri et al., 2008 [10] (28)	Penicillin	Emergency CS	Hypoxia and acidosis. Encephalopathy.
		Göktaş et al., 2010 [51] (36)	Sulbactam, ampicillin	Emergency CS	Favorable outcome.

CS: Cesarean section; LI: labor induction after fetal death.

## Supplementary Materials

The following are available online at https://www.mdpi.com/article/10.3390/jpm11111060/s1, Table S1: Studies included in the analysis and reported allergen for anaphylaxis; Table S2: Time to the occurrence of anaphylaxis and between contact to the onset of the first symptom, comorbidities, mode of delivery at the admission and actual mode of delivery; Table S3: Maternal symptomatology, treatment, laboratory tests, and outcomes.
