# Supplementary material for: Severe Anaphylaxis in Pregnancy: A Systematic Review of Clinical Presentation to Determine Outcomes
Source: J Pers Med. 2021 Oct 22;11(11):1060. doi: 10.3390/jpm11111060 (PMC8623240; doi:10.3390/jpm11111060)
Supplement: Supplementary file 1 [file jpm-11-01060-s001.zip › jpm-1318917-supplementary.pdf]

Supplementary material

Table S1: Studies included in the analysis and reported allergen for anaphylaxis

Number	Studies references in chronological apparition between 1985-2021	Country	Number of cases	Allergen	Gestational age (weeks)	Score for method quality evaluation*
1	Gallagher JS. Anaphylaxis in pregnancy. Obstet Gynecol. 1988 Mar;71(3 Pt 2):491-3.	USA	1	Penicillin	36-37(preterm birth)	6
2	Jorrot JC, Mercier F, Pecquet C, Jacquinot P, Conseiller C. Choc anaphylactique peropératoire au latex [Peroperative anaphylactic shock caused by latex]. Ann Fr Anesth Reanim. 1989;8(3):278-9. French. doi: 10.1016/s0750-7658(89)80119-4.	France	1	Latex	39(term)	2
3	Anderson TH, Hindsholm KB, Fallingborg J. Severe complication to phytomenadione after intramuscular injection in woman in labor. Case report and review of literature. Acta Obstet Gynecol Scand. 1989;68(4):381-2. doi: 10.3109/00016348909028678.	Denmark	1	Phytomenadione (oral)	term	2
4	Heim K, Alge A, Marth C. Anaphylactic reaction to ampicillin and severe complication in the fetus.	Austria	1	Ampicillin	term	4

	Lancet. 1991 Apr 6;337(8745):859-60. doi: 10.1016/0140-6736(91)92574-l.					
5	Laurent J, Malet R, Smiejan JM, Madelenat P, Herman D. Latex hypersensitivity after natural delivery. J Allergy Clin Immunol. 1992 Mar;89(3):779-80. doi: 10.1016/0091-6749(92)90389-j.	France	1	Latex	term	3
6	Powell JA, Maycock EJ. Anaphylactoid reaction to ranitidine in an obstetric patient. Anaesth Intensive Care. 1993 Oct;21(5):702-3. doi: 10.1177/0310057X9302100539.	New Zealand	1	Ranitidine	term	2
7	Edmondson WC, Skilton RW. Anaphylaxis in pregnancy--the right treatment? Anaesthesia. 1994 May;49(5):454-5. doi: 10.1111/j.1365-2044.1994.tb03501.x.	UK ,Wales	1	Suxamethonium	36(preterm)	4
8	Konno R, Nagase S. Anaphylactic reaction to cefazolin in pregnancy. J Obstet Gynaecol (Tokyo 1995). 1995 Dec;21(6):577-9. doi: 10.1111/j.1447-0756.1995.tb00915.x	Japan	1	Cefazolin	36(preterm)	3
9	Stewart PD, Bogod D. Latex anaphylaxis during late pregnancy. Int J Obstet Anesth. 1995 Jan;4(1):48-50.	UK	1	Rubber (Foley catheter)	33(preterm)	3

	doi: 10.1016/0959-289x(95)82443-e.					
10	Diaz T, Martínez T, Antépara I, Usandizaga JM, López Valverde M, Jáurequi I. Latex allergy as a risk during delivery. Br J Obstet Gynaecol. 1996 Feb;103(2):173-5. doi: 10.1111/j.1471-0528.1996.tb09672.x	Spain	3	Latex	term	3
11	Rae SM, Milne MK, Wildsmith JA. Anaphylaxis associated with, but not caused by, extradural bupivacaine. Br J Anaesth. 1997 Feb;78(2):224-6. doi: 10.1093/bja/78.2.224.	UK, Scotland	1	Latex	term	2
12	Rizk DE, Mensah-Brown E, Lukic M. Placental abruption and intrauterine death following an ant sting. Int J Gynaecol Obstet. 1998 Oct;63(1):71-2. doi: 10.1016/s0020-7292(98)00129-5.	United Arab Emirates	1	Ant sting (Pachycondyla sennaarensis or Samsun)	40 (term)	2
13	Jensen-Jarolim E, Reider N, Fritsch R, Breiteneder H. Fatal outcome of anaphylaxis to camomile-containing enema during labor: a case study. J Allergy Clin Immunol. 1998 Dec;102(6 Pt 1):1041-2. doi: 10.1016/s0091-6749(98)70345-8	Austria	1	Extract of camomile flowers	term	3
14	Cole DS, Bruck LR. Anaphylaxis after laminaria insertion. Obstet Gynecol. 2000 Jun;95(6 Pt	UK	1	Laminaria	21(Abortion induction)	3

	2):1025. doi: 10.1016/s0029-7844(00)00785-7.					
15	Stannard L, Bellis A. Maternal anaphylactic reaction to a general anaesthetic at emergency caesarean section for fetal bradycardia. BJOG. 2001 May;108(5):539-40. doi: 10.1111/j.1471-0528.2001.00107.x.	UK	1	Suxamethonium	38(term)	2
16	Eckhout GV Jr, Ayad S. Anaphylaxis due to airborne exposure to latex in a primigravida. Anesthesiology. 2001 Oct;95(4):1034-5. doi: 10.1097/00000542-200110000-00040.	USA	1	Latex	32 (consultation for preterm labor)	2
17	Shingai Y, Nakagawa K, Kato T, Fujioka T, Matsumoto T, Kihana T, Noda K, Mori T. Severe allergy in a pregnant woman after vaginal examination with a latex glove. Gynecol Obstet Invest. 2002;54(3):183-4. doi: 10.1159/000067888.	Japan	1	Latex	38 (term)	2
18	Knowles SR, Djordjevic K, Binkley K, Weber EA. Allergic anaphylaxis to Laminaria. Allergy. 2002 Apr;57(4):370. doi: 10.1034/j.1398-9995.2001.1n3459.x.	Canada	1	Laminaria digitata	Abortion induction	4
19	Gei AF, Pacheco LD, Vanhook JW, Hankins GD. The use of a continuous infusion of epinephrine for anaphylactic shock during	USA	1	Ampicillin	40(term)	2

	labor. Obstet Gynecol. 2003 Dec;102(6):1332-5. doi: 10.1016/s0029-7844(03)00167-4. Erratum in: Obstet Gynecol. 2004 Apr;103(4):799.					
20	Kim SH, Chang YH, Kim WK, Kim YK, Cho SH, Kim YY, Min KU. Two cases of anaphylaxis after laminaria insertion. J Korean Med Sci. 2003 Dec;18(6):886-8. doi: 10.3346/jkms.2003.18.6.886.	Korea	1	Laminaria	Abortion induction	3
21	Berardi A, Rossi K, Cavalleri F, Simoni A, Aguzzoli L, Masellis G, Ferrari F. Maternal anaphylaxis and fetal brain damage after intrapartum chemoprophylaxis. J Perinat Med. 2004;32(4):375-7. doi: 10.1515/JPM.2004.070.	Italia	1	Ampicillin	37 (preterm)	1
22	Cuciti C, Mayer DC, Arnette R, Spielman FJ. Anaphylactoid reaction to intravenous sodium ferric gluconate complex during pregnancy. Int J Obstet Anesth. 2005 Oct;14(4):362-4. doi: 10.1016/j.ijoa.2005.05.001.	USA	1	Sodium ferric gluconate complex	38(term)	2
23	Vatsgar TT, Ingebrigtsen O, Fjose LO, Wikstrøm B, Nilsen JE, Wik L. Cardiac arrest and resuscitation with an automatic mechanical chest compression device (LUCAS) due to anaphylaxis of a woman	Norway	1	Dextran	24 (preterm)	2

	receiving caesarean section because of pre-eclampsia. Resuscitation. 2006 Jan;68(1):155-9. doi: 10.1016/j.resuscitation.2005.06.001.					
24	Turillazzi E, Greco P, Neri M, Pomara C, Riezzo I, Fineschi V. Anaphylactic latex reaction during anaesthesia: the silent culprit in a fatal case. Forensic Sci Int. 2008 Jul 18;179(1):e5-8. doi: 10.1016/j.forsciint.2008.03.021.	Italy	1	Latex	38(term)	1
25	Khan R, Anastasakis E, Kadir RA. Anaphylactic reaction to ceftriaxone in labour. An emerging complication. J Obstet Gynaecol. 2008 Oct;28(7):751-3. doi: 10.1080/01443610802260595.	UK	1	Ceftriaxone	38	2
26	Chaudhuri K, Gonzales J, Jesurun CA, Ambat MT, Mandal-Chaudhuri S. Anaphylactic shock in pregnancy: a case study and review of the literature. Int J Obstet Anesth. 2008 Oct;17(4):350-7. doi: 10.1016/j.ijoa.2008.05.002.	USA	1	Penicillin	40(term)	2
27	Delaunay F, Blasco V. Choc anaphylactique au latex en cours de césarienne: à propos de deux cas survenus en Guadeloupe [Latex induced anaphylactic shock during	Guadeloupe	1<br><br>1	Latex<br><br>Latex	Term<br><br>36 (preterm)	4

	caesarean section: two cases fro Guadeloupe]. Ann Fr Anesth Reanim. 2008 Dec;27(12):1023-5. French. doi: 10.1016/j.annfar.2008.10.007 .					
28	Pant D, Vohra VK, Pandey SS, Sood J. Pulseless electrical activity during caesarean delivery under spinal anaesthesia: a case report of severe anaphylactic reaction to Syntocinon. Int J Obstet Anesth. 2009 Jan;18(1):85-8. doi: 10.1016/j.ijoa.2008.09.004.	India	1	Oxytocin	Term	7
29	Rocchiccioli C, Aldea R, Guinnepain MT, Fischler M. Choc anaphylactique dû à la succinylcholine compliqué de rhabdomyolyse [Anaphylactic shock from succinylcholine, complicated by a rhabdomyolysis]. Ann Fr Anesth Reanim. 2009 Sep;28(9):787-9. French. doi: 10.1016/j.annfar.2009.06.024 .	France	1	Suxamethonium	12 (abortion)	3
30	Karri K, Raghavan R, Shahid J. Severe Anaphylaxis to Volplex, a Colloid Solution during Cesarean Section: A Case Report and Review. Obstet Gynecol Int. 2009;2009:374791. doi: 10.1155/2009/374791	UK	1	Volplex	Term	2

31	Sleth JC, Lafforgue E, Cherici O, Nagy P. Choc anaphylactique au cours de la grossesse à terme. A propos de deux cas et revue de la littérature [Anaphylaxis in terminal pregnancy: two case studies and review of the literature]. Ann Fr Anesth Reanim. 2009 Sep;28(9):790-4. French. doi: 10.1016/j.annfar.2009.06.023 .	France	1<br>1	37 (preterm)<br>38(term)	Suxamethonium<br>Amoxicillin	6
32	Göktaş U, Katı İ, Tekin M, Güneş Y. A Pregnant Developed Cardiac Arrest Due to Anaphylaxis. Eur J Gen Med. 2010;7(1), 98-100. doi : 10.29333/ejgm/82827	Turkey	1	36 (preterm)	Sulbactam Ampicillin	3
33	Mishra A, Dave N, Viradiya K. Fatal anaphylactic reaction to iron sucrose in pregnancy. Indian J Pharmacol. 2013 Jan-Feb;45(1):93-4. doi: 10.4103/0253-7613.106446.	India	1	7 month(preterm)	Iron sucrose	1
34	Liccardi G, Bilò MB, Mauro C, Salzillo A, Piccolo A, D'Amato M, D'Amato G. Oxytocin: a likely underestimated risk for anaphylactic reactions in delivering women sensitized to latex. Ann Allergy Asthma Immunol. 2013 Jun;110(6):465-6. doi: 10.1016/j.anai.2013.03.014.	Italy	1<br>1	37(preterm)<br>36(preterm)	Oxytocin<br>Oxytocin	2
35	Béné J, Alarcon P, Faucon M, Auffret M, Delfosse F,	France	1	9 (abortion induction)	Misoprostol	3

	Becker T, De Zorzi S, Gautier S. Anaphylactic shock after misoprostol in voluntary termination of pregnancy—a case report. Eur J Obstet Gynecol Reprod Biol. 2014 Nov;182:260-1. doi: 10.1016/j.ejogrb.2014.09.012.					
36	Schoen C, Campbell S, Maratas A, Kim C. Anaphylaxis to buccal misoprostol for labor induction. Obstet Gynecol. 2014 Aug;124(2 Pt 2 Suppl 1):466-468. doi: 10.1097/AOG.0000000000000268.	USA	1	41(postterm)	Misoprostol	3
37	Truong HT, Browning RM. Anaphylaxis-induced hyperfibrinolysis in pregnancy. Int J Obstet Anesth. 2015 May;24(2):180-4. doi: 10.1016/j.ijoa.2014.12.009.	Australia	1	19 (cervical cerclage)	Suxamethonium	Cerclaj 19sapt
38	Yamaoka M, Deguchi M, Ninomiya K, Kurasako T, Matsumoto M. A suspected case of rocuronium-sugammadex complex-induced anaphylactic shock after cesarean section. J Anesth. 2017 Feb;31(1):148-151. doi: 10.1007/s00540-016-2280-4	Japan	1	term	Rocuronium-Sugammadex complex	2
39	Jeon HJ, Ryu A, Min J, Kim NS. Maternal anaphylactic shock in pregnancy: A case report. Medicine (Baltimore). 2018 Sep;97(37):e12351. doi:	Korea	1	term	Cefotetan	2

	10.1097/MD.0000000000012 351.					
40	Rial Prado MJ et al Doxylamine Allergy in a Pregnant Woman: Suitability of the Basophil Activation Test. J Investig Allergol Clin Immunol 2018 , 28(6):433-34	Spain	1	First trimester	Pyridoxine	1
41	Takahashi M, Hotta K, Inoue S, Takazawa T, Horiuchi T, Igarashi T, Takeuchi M. Mepivacaine-induced anaphylactic shock in a pregnant woman undergoing combined spinal and epidural anesthesia for cesarean delivery: a case report. JA Clin Rep. 2019 Dec 19;5(1):84. doi: 10.1186/s40981-019-0302-6.	Japan	1	term	Mepivacaine	2
42	McQuade M, Barbour K, Betstadt S, Harrington A. Intubation and intensive care after laminaria anaphylaxis in second-trimester abortion. Am J Emerg Med. 2020 Jan;38(1):163.e1-163.e2. doi: 10.1016/j.ajem.2019.158409.	USA	1	21+5 (induced abortion	laminaria	Trim 2

*score was calculated based on methodological quality and synthesis of case reports from Murad et al. [27]

Table S2 : Time to the occurrence of anaphylaxis and between contact to the onset of the first symptom , comorbidities, mode of delivery at the admission and actual mode of delivery .

Studies	Case number	Allergen	Reason for the hospital / delivery room admission	Time to the onset of the first symptom of anaphylaxis	Gestational age (weeks)	Stage of labor / CS when anaphylaxis occurred	Mode of delivery
Gallagher . 1988 [28]	1	Penicillin	Labor PRM,corioamnionite	5 minutes	36-37	Fully dilatated	V
Jorrot 1989 [29]	2	Latex (and oxytocin)	Scheduled CS for scarred uterus	10 minutes, during CS , after D	39	Before labor	Scheduled CS
Anderson 1989[30]	3	Phytomenadione (oral)	Labor	15 minutes	term	During labor	Emergent CS
Heim 1991[31]	4	Ampicillin	Labor	5 minutes	term	During labor	Emergent CS
Laurent 1992 [32]	5	Latex	Labor induction	Few minutes	term	After birth	V
Powell 1993 [33]	6	Ranitidine	Labor -4 cm , fetal distress and emergent CS	Immediately	term	4 cm	V
Edmondson 1994[34]	7	Suxamethonium	Third trimester , pilonidal abscess incision , general anesthesia	10 minutes	36	No in labor	Emergent CS
Konno 1995[35]	8	Cefazolin	Labor	5 minutes	36(preterm)	Labor	Emergent CS
Stewart 1995 [36]	9	Rubber (Foley catheter)	Scheduled CS for preeclampsia	5 minutes	33(preterm)	Before CS	Emergent CS
Diaz 1996[37]	10	Latex	Labor	Immediately	term	Labor	Emergent CS
	11		Scheduled CS			CS	? CS

	12		Scheduled CS			CS	?CS
Rae, 1997[38]	13	Latex	Scheduled CS	40-50 minutes	term	After CS	Scheduled CS
Rizk , 1998[39]	14	Ant sting (Pachycondyla sennaarensis or Samsun)	Anaphylaxis	Immediately	40 (term)	Before labor / stillbirth	-
Jensen-Jarolim , 1998 [40]	15	Extract of camomile flowers	Labor induction	5 minutes	term	Before labor	Emergent CS
Cole DS, 2000[41]	16	Laminaria	Therapeutic abortion	5 minutes	21(Abortion induction)	-	-
Stannard L, 2001[42]	17	Suxamethonium	Labor	Immediately	38(term)	After APD , CS for fetal bradycardia	Emergent CS before anaphylaxis
Eckhout, 2001[43]	18	Latex	Consultation for uterine contractions	immediately	32 (consultation for preterm labor)	Third trimester	V (at term)
Shingai 2002[44]	19	Latex	Before labor	minutes	38 (term)	Before labor	Emergent CS
Knowles 2002[45]	20	Laminaria digitata	Therapeutic abortion	30 minutes	Abortion induction	-	-
Gei , 2004 [46]	21	Ampicillin	Labor, MR from 15 hours	Immediately	40(term)	Labor	V
Kim , 2003 [47]	22	Laminaria	Therapeutic abortion	30 minutes	Abortion induction	-	-
Berardi 2004[9]	23	Ampicillin	Labor, MR from 12 hours	Immediately	37 (preterm)	Labor	Emergent CS
Cuciti , 2005 [48]	24	Sodium ferric gluconate complex	Maternal anemia	Shortly	38(term)	Before labor	V (after one week)

Vatsgar, 2006 [49]	25	Dextran	Scheduled CS for severe preeclampsia	Immediately	24 (preterm)	Before CS	Emergent CS
Turillazzi , 2008[50]	26	Latex	Scheduled CS	10 minutes	38(term)	After delivery of the baby	Scheduled CS
Khan , 2008[51]	27	Ceftriaxone	Labor, chorioamnionitis	Immediately	38	Labor	Emergent CS
Chaudhuri, 2008[20]	28	Penicillin	Labor, MR	Few minutes	40(term)	Labor	Emergent CS
Delaunay , 2008[52]	29	Latex	Scheduled CS for twins	10 minutes	Term	After delivery of the baby	Scheduled CS
	30	Latex	Scheduled CS for preeclampsia	Minutes	36 (preterm)	M	M
Pant, 2009 [53]	31	Oxytocin	Scheduled CS for twins	Minutes	Term	After delivery of the babies	Scheduled CS
Rocchiccioli , 2009[54]	32	Suxamethonium	Abortion induction , general anesthesia	Minutes	12 (abortion)	-	-
Karri , 2009[55]	33	Volplex	Scheduled CS for failed induction of labor	Minutes	Term	M	M
Sleth , 2009[15]	34	Suxamethonium	Third trimester, condylomas	Immediately	37	Before labor	Emergent CS
	35	Amoxicillin	Labor	Minutes	38	Labor	Emergent CS
Göktaş , 2010 [56]	36	Ampicillin	Labor, chorioamnionitis	1 minute	36 (preterm)	Labor	Emergent CS

Mishra,2013 [57]	37	Iron sucrose	Maternal anemia, Third trimester	Few minutes	7 month(preterm)	Third trimester, maternal death	-
Liccardi , 2013[58]	38	Oxytocin	Scheduled CS for Preeclampsia	minutes	37(preterm)	M	M
	39	Oxytocin	Scheduled CS for placenta praevia	Minutes	36(preterm)	M	M
Béné , 2014[59]	40	Misoprostol	9weeks (abortion induction)	minutes	-	-	-
Schoen , 2014[60]	41	Misoprostol	Labor induction	M	Postterm	Before labor	Emergent CS
Truong ,2015[61]	42	Suxamethonium	Scheduled cervical cerclage , general anesthesia	Minutes	19 (cervical cerclage)	Second trimester, stillbirth	-
Yamaoka , 2017 [62]	43	Rocuronium-Sugammadex complex	Scheduled CS for placenta praevia, general anesthesia	10 minutes after CS	Term	CS	Scheduled CS
Jeon , 2018 [63]	44	Cefotetan	Scheduled CS for scarred uterus	Immediate	Term	CS	Scheduled CS
Rial 2018 [64]	45	Pyridoxine	Hyperemesis gravidorum	Less than 60 minutes	First trimester	M	M
Takahashi, 2019[65]	46	Mepivacaine	Scheduled CS for twins, epidural anesthesia	Immediate	Term	CS	Emergent CS
McQuade, 2020[66]	47	Laminaria	Therapeutic abortion	Immediate	21+5 (induced abortion	-	-

MR: premature rupture of membrane ; CS: Cesarean section; V: vaginal delivery ; D: delivery of the baby; APD: peridural anesthesia ; M:missing

Table S3. Maternal symptomatology, treatment, laboratory tests, and outcomes.

Study/year	Case number	Clinical signs and symptoms	Allergen potential/ culprit	Treatment	Maternal outcome		Biochemical tests	Skin tests at distance
					Evolution[a]	Discharge,[b] days		
Gallagher et al., 1988[28]	1	Burning, itching, diffuse leg edema, laryngeal spasm, hypotension	Penicillin	Diphenhydramine, methylprednisolone, adrenaline, colloid	Few days	4	M	M
Jorrot et al., 1989[29]	2	Sweating, agitation, temporal disorientation, rash generalized, palpebral edema, hypotension, tachycardia	Oxytocin and latex /Latex	Adrenaline	NS	8	+	+
Anderson, 1989 [30]	3	Pruritus, hypotension, dyspnea, edema	Phyto menadione (oral)	Adrenaline	NS	NS	+	+
Heim et al., 1991[31]	4	Itching, burning, nausea	Ampicillin	Glucocorticoid Calcium Antihistamine	NS	NS	M	M
Laurent et al., 1992[32]	5	Itching, nasal irritation, sneezing, rash cutaneous, glottic edema, bronchospasm, maternal cardiorespiratory shock	Oxytocin and latex /Latex	Epinephrine Complex resuscitation	2 h	NS	+	+
Powell et al., 1993[33]	6	Horrible taste, dyspnea, laryngeal spasm, hypotension, tachycardia, edema	Ranitidine	Promethazine Hydrocortisone	3 days	NS	M	+
Edmondson et al., 1994[34]	7	Hypoxemia, rash, hypotension, tachycardia	Suxamethonium, fentanyl / Suxamethonium	Hydrocortisone Adrenaline	NS	NS	+	+
Konno et al., 1995[35]	8	Burning, itching, nausea, edema,	Cefazolin	Ephedrine Methylprednisolone	2 days	13	M	M

		hypotension, nausea						
Stewart et al., 1995[36]	9	Dyspnea, bronchospasm, cyanosis, cardiorespiratory shock, edema	Rubber	Ventilated and intubated<br>External cardiac massage<br>Adrenaline<br>Gelofusin	1 day	10	+	+
Diaz et al., 1996[37]	10	Skin rash and pruritus, edema angioneurotic, bronchospasm, hypotension	Latex	Adrenaline<br>Antihistaminic<br>Corticosteroids	Hours	NS	+	+
	11	Urticaria, edema angioneurotic, bronchospasm, hypotension			NS	NS	+	+
	12	Urticaria, edema angioneurotic, bronchospasm, hypotension			NS	NS	+	+
Rae et al., 1997[38]	13	Hypotension, cyanosis, dyspnea, cardiovascular shock	Rubber, bupivacaine, and hibitane / Latex	Adrenaline<br>Colloids	NS	NS	M	+
Rizk et al., 1998[39]	14	Dyspnea, hypotension, tachycardia	Ant sting	Chlorpheniramine<br>Methylprednisolone<br>Epinephrine	NS	NS	M	+
Jensen-Jarolim et al., 1998[40]	15	Urticaria, laryngeal edema hypotension, tachycardia	Extract of chamomile flowers	Corticosteroids<br>Antihistaminic<br>Effortil	> 1 day	NS	+	+
Cole et al., 2000[41]	16	Dyspnea, urticaria	Laminaria	Antihistamine, corticosteroids	1 day	~7	NA	NA
Stannard et al., 2001[42]	17	Cardiorespiratory shock, hypoxemia, bronchospasm, supraventricular tachycardia	Suxamethonium	Adrenaline<br>Colloid infusion<br>Chlorpheniramine<br>Tracheostomy	NS	~28	+	+
Eckhout et al., 2001[43]	18	Dyspnea, wheezing, generalized rash urticarial, hypotension	Latex	Diphenhydramine<br>Hydrocortisone acetate	20 min	NS	M	+

Shingai et al., 2002[44]	19	Urticarial rash, dyspnea	Latex	Hydrocortisone<br>Epinephrine	2 h	NS	M	+
Knowles et al., 2002[45]	20	Urticaria, dyspnea, edema, coughing	Laminaria digitata	Diphenhydramine	Few hours	NS	NA	NA
Gei et al., 2004[46]	21	Dyspnea, rash, hypotension, tachycardia	Ampicillin	Diphenhydramine<br>Crystalloid solutions<br>Epinephrine	24 h	2 d	M	M
Kim et al., 2003[47]	22	Dyspnea, hypotension	Laminaria	Epinephrine<br>Methylprednisolone	NS	NS	NA	NA
Berardi et al., 2004 [9]	23	Pruritus, urticaria, hypotension	Ampicillin	Steroids<br>Antihistamine<br>Etilefrine	NS	NS	M	M
Cuciti et al., 2005[48]	24	Edema, hypotension, tachycardia, ventricular bigeminism	Sodium ferric gluconate complex	Diphenhydramine<br>Epinephrine<br>Ranitidine<br>Dexamethasone<br>Crystalloids	24 h	NS	M	M
Vatsgar et al., 2006[49]	25	Bronchospasm, cardiorespiratory shock	Dextran	Adrenaline<br>Noradrenaline<br>Mechanical chest compression	Extubated after 32 h	NS	+	M
Turillazzi et al., 2008[50]	26	Bronchospasm, hypotension, DIC	Latex	Vasoactive drugs<br>Complex reanimation	Maternal death	-	+	+
Khan et al., 2008[51]	27	Dyspnea, urticaria, edema, tachycardia, hypotension	Ceftriaxone	Colloid and crystalloid solutions<br>Chlorpheniramine<br>Hydrocortisone<br>Adrenaline	NS	6	M	M
Chaudhuri et al., 2008[20]	28	Erythema, hypotension, tachycardia	Penicillin	Diphenhydramine<br>Ephedrine<br>Epinephrine	24 h	2	M	M
Delaunay et al., 2008[52]	29	Urticaria, cardiovascular shock, bronchospasm	Latex, bupivacaine, oxytocin, cefazolin /Latex	Adrenaline<br>Methylprednisolone	24 h	NS	+	+
	30	Stop cardiorespiratory		External cardiac massage, adrenaline	72 h	NS	+	+

Pant et al., 2009[53]	31	Hypotension, hypoxemia, rash, pruritus, laryngospasm, sinus tachycardia, cardiorespiratory shock	Methylergometrine, oxytocin /Oxytocin	Pentastarch Hydrocortisone External cardiac massage Epinephrine, mannitol	24 h	NS	+	+
Rocchicci oli et al., 2009[54]	32	Cutaneous rash, hypotension, tachycardia, cardiorespiratory shock	Suxamethonium	Colloids, adrenaline, methylprednisolone	NS	NS	+	+
Karri et al., 2009[55]	33	Dyspnea Hypotension	Volplex	Adrenaline Hydrocortisone Chlorpheniramine	NS	NS	+	M
Sleth et al., 2009[15]	34	Cardiorespiratory shock	Suxamethonium, propofol /Suxamethonium	Complex reanimation measures	NS	NS	+	+
	35	Erythema, hypotension, tachycardia, cardiovascular shock	Amoxicillin	Adrenaline Complex reanimation measures	NS	NS	+	+
Göktaş et al., 2010[56]	36	Hypotension, tachycardia	Sulbactam, ampicillin	Ephedrine Phenylephrine Adrenaline	2 h	NS	M	M
Mishra et al., 2013[57]	37	Hypotension, urticaria, cyanosis, cardiovascular shock	Iron sucrose	Complex reanimation measures	Maternal death	-	M	M
Liccardi et al., 2013[58]	38	Generalized rush and urticaria-angioedema, macroglossia, intraoral and laryngeal angioedema, profound hypotension (60/40 mmHg)	Latex, bupivacaine, oxytocin/ Oxytocin	Adrenaline Hydrocortisone Antihistamine Complex reanimation measures	NS	NS	+	+
	39	Generalized and facial rush/urticaria-angioedema,	Latex, bupivacaine, oxytocin/ Oxytocin	Complex reanimation measures			+	+

		dyspnea, hypotension						
Béné et al., 2014[59]	40	Hypoxemia, shock cardiorespiratory, urticaria	Misoprostol	Adrenaline, hydroxyethyl starch, betamethasone, methylprednisolone, dexchlorphenyramine , complex reanimation measures	NS	2	+	NA
Schoen et al., 2014[60]	41	Pruritic, urticaria, dyspnea, laryngeal edema	Misoprostol	Diphenhydramine Ephedrine	Extubated after 24 h	3	+	NA
Truong et al., 2015[61]	42	Stop cardiorespiratory	Suxamethonium	Complex reanimation measures	Extubated after 24 h	NS	+	+
Yamaoka et al., 2017[62]	43	Dyspnea, hypotension, unconscious	Rocuronium-sugammadex complex	Complex reanimation measures, ephedrine , hydrocortisone, famotidine, chlorpheniramine maleate	NS	NS	+	+
Jeon, 2018 [63]	44	Pruritus, urticarial rash, dyspnea, hypotension, tachycardia, hypoxemia	Cefotetan	Antihistamine Hydrocortisone	NS	NS	M	+
Rial et al., 2018[64]	45	Hypotension, dyspnea, generalized pruritus, urticaria	Pyridoxine	Hydrocortisone Dexchlorpheniramine	2 h	NS	+	+
Takahashi et al., 2019[65]	46	Hypotension, tachycardia, generalized edema	Mepivacaine	Phenylephedrine Methylprednisolone	NS	NS	+	+
McQuade et al., 2020[66]	47	Dyspnea, cutaneous rash,	Laminaria	Epinephrine, diphenhydramine , dexamethasone	NS	1	NA	NA

[a]Time to normal evolution; [b]Time to discharge home in days. NS, not clearly specified; NA, not attributed; M, missing; DIC, disseminated intravascular coagulation.
